# Role of beta-blockers in reducing post-operative atrial fibrillation following cardiac surgery

**DOI:** 10.6026/973206300210774

**Published:** 2025-04-30

**Authors:** Kumari Sneha, Kalyan Kumar Saha, Srirupa Mandal, Samrat Smrutiranjan Sahoo, Vinod Kumar, Mukesh Kumar, Sommya Kumari

**Affiliations:** 1 Department of Anaesthesia, AIIMS, Kalyani, West Bengal, India; 2 Department of Cardiology, AIIMS, Kalyani, West Bengal, India; 3 Department of Orthopedics, AIIMS, Kalyani, West Bengal, India; 4 Department of Urology, AIIMS, Kalyani, West Bengal, India; 5 Department of Dentistry, AIIMS, Kalyani, West Bengal, India; 6 Department of Dentistry, AIIMS Deoghar, Jharkhand, India

**Keywords:** Beta-blockers, postoperative atrial fibrillation, cardiac surgery

## Abstract

The evaluation of nine clinical investigations which studied 2,533 patients demonstrates beta-blockers reduce postoperative atrial fibrillation (POAF) after cardiac surgical procedures effectively. It is known that beta-blockers successfully decreased the occurrence of postoperative atrial fibrillation 11.1% to 52.0%. Differences in patient characteristics and drug regimen make it difficult to determine the properly adjusted beta-blocker treatment for ideal results. Thus, beta-blocker therapy for reducing postoperative atrial fibrillation cases is relevant yet additional standard protocols must be created.

## Background:

Postoperative atrial fibrillation (POAF) is a prevalent complication following cardiac surgery, with reported incidence rates ranging from 20% to 50%, depending on the surgical procedure, definition, and diagnostic tools employed [1, 2]. It is associated with increased risks of morbidity and mortality due to hemodynamic instability, heart failure, thromboembolism, and bleeding complications related to anticoagulation [3]. The pathophysiologic mechanisms underlying POAF are multifactorial, involving postoperative inflammatory responses, catecholamine surges, autonomic nervous system imbalance, changes in intravascular volume status, and substrates created by the surgical procedure itself, including alterations in cardiac structures and perioperative ischemia [4, 5, 6-7].

Postoperative atrial fibrillation (POAF) poses a significant challenge in postoperative management, leading to increased complications such as postoperative stroke and prolonged hospital stays [8]. According to existing research; beta-blockers (β-blockers) have emerged as a universally recognized prophylactic agent, effective in reducing the incidence of POAF [8]. Current clinical guidelines recommend the use of β-blockers as a standard therapy for prophylactic management of POAF [9, 10]. Therefore, it is of interest to evaluate the role of beta-blockers in reducing postoperative atrial fibrillation (POAF) following cardiac surgery. The effectiveness of beta-blockers in preventing POAF so as to identify the optimal beta-blocker regimen is of interest.

## Materials and Methods:

The inclusion criteria were framed as per internationally standardized PICOS framework, as recommended by PRISMA guidelines:

## Participants/population:

The study population included patients who underwent cardiac surgery.

## Intervention:

Patients who underwent cardiac surgery were diagnosed with atrial fibrillation.

## Comparator(s)/control:

Studies of any of the above-mentioned interventions were included, including studies with no comparator group.

## Outcome:

The key outcomes consider were assessment of role of beta-blockers in reducing postoperative atrial fibrillation following cardiac surgery. Primary outcomes of the study were to determine role of beta-blockers in reducing postoperative atrial fibrillation following cardiac surgery and secondary outcome was assessment of any reported associated factors that increase the risk of atrial fibrillation among patients who underwent cardiac surgery.

## Study design:

The review included all types of experimental studies, observational studies and case series which have reported the outcomes of the above-mentioned role of beta-blockers in reducing postoperative atrial fibrillation following cardiac surgery.

## Inclusion criteria:

Studies conducted anywhere in the world and articles published after 2010 through April 2023 was included in the study.

Only those studies published in English language, academic peer-reviewed journals were included in the review.

## Exclusion criteria:

Case studies were excluded from the study. Studies conducted on animals were excluded from the study.

## Literature search:

A systematic literature search was performed in PubMed, Embase, clinical trial.gov, google scholar and Cochrane Library through December 2024 in the English language by two independent authors using a structured search strategy. The searches were screened by the references of selected articles to find those that did not appear in the search databases. Additional references were not obtained by free internet search from Google as the number of studies was large. The detail search strategy is given in Table 1.

## Process of screening and selection of articles:

All the citations along with the title and abstract were added to a specified endnote library and final list of studies to be screened for inclusion in the study was prepared by removing the duplicates. Two researchers carefully screened the articles by assessment of the title and thorough reading the abstracts to shortlist the studies which are likely to satisfy the inclusion criteria of the review. Attempts were made to obtain full-text articles for all these shortlisted studies, and thorough assessment was done for the satisfaction of inclusion and exclusion criteria. Studies not satisfying inclusion criteria were excluded further. The list of excluded studies and the reasons for exclusion were presented in the "characteristics of excluded studies" table. "PRISMA flow chart" was used to clearly represent the screening and selection process (Figure 1 - see PDF).

## Data extraction:

Data was thoroughly read through and were extracted from included studies was extracted manually on to a structured data extraction form. The number of patients *i.e.*, incidence of patients with atrial fibrillation among diabetic patients, any reported gender prevalence as well as any associated factors that increase the risk of atrial fibrillation among diabetic individuals were reported.

## Risk of bias in individual studies:

The methodological quality of studies included in the systemic review was assessed according to Fowkes and Fulton quality assessment [10].

## Outcome:

A total of 9 studies that fulfilled inclusion criteria were included in this analysis, which investigated the role of beta-blockers in reducing postoperative atrial fibrillation following cardiac surgery (Table 1). These studies were published between 2010 and 2024, indicating a recent and ongoing interest in the topic of beta-blockers and postoperative atrial fibrillation. These studies were conducted across various countries, including the Netherlands, Europe, Korea, Japan, Turkey, China, Australia and Finland. The study designs were diverse, comprising retrospective studies; cohort studies, prospective randomized controlled studies, and randomized prospective equivalence open-label multicenter studies. The number of participants in the study groups ranged from 150 to 614 patients. The mean age of the participants varied from 56.1 to 74.5 years. The gender distribution was reported in some studies, with males ranging from 97 to 342 and females ranging from 53 to 272. Overall, these studies aimed to investigate the efficacy of beta-blockers in reducing postoperative atrial fibrillation following cardiac surgery, with varying study designs, sample sizes, and patient populations.

The studies examined various operative procedures (Table 2), including coronary artery bypass grafting (CABG), valve surgery, aortic valve replacement, and surgical revascularization. Some studies also investigated combined procedures, such as CABG and valve surgery.

## Postoperative atrial fibrillation (POAF):

The incidence of POAF varied across studies, ranging from 11.1% to 52.0% (Table 2). Some studies reported the number of patients who developed POAF, while others provided the percentage of patients who experienced this complication. The timing of POAF also varied, with some studies reporting the mean time to POAF onset.

## Drug regimen:

The studies examined various beta-blocker regimens, including preoperative, postoperative, or both pre- and postoperative administration. The specific beta-blockers used included metoprolol, carvedilol, propranolol, bisoprolol, nebivolol, atenolol, and landiolol. The dosages and frequencies of administration also varied across studies. Some studies compared beta-blockers with other antiarrhythmic agents, such as amiodarone.

The drug regimens used in the studies varied in terms of the type of beta-blocker, dosage, frequency, and duration of administration.

## Types of beta-blockers:

The studies used various types of beta-blockers, including:

[1] Metoprolol: Used in several studies, including Onk *et al.* (2015) [14], Cheng *et al.* (2014) [15], Skiba *et al.* (2013) [16] and Halonen *et al.* (2010) [18].

[2] Carvedilol: Used in Kim *et al.* (2020) [3] with a dosage of 12.5mg (3.0-25.0).

[3] Propranolol: Used in Kim *et al.* (2020) [3] with a dosage of 20.0mg (20.0-40.0).

[4] Bisoprolol: Used in Kim *et al.* (2020) [3] with a dosage of 2.5mg (1.25-5.0).

[5] Nebivolol: Used in Kim *et al.* (2020) [3] with a dosage of 2.5mg (1.25-5.0).

[6] Atenolol: Used in Kim *et al.* (2020) [3] with a dosage of 50mg (25.0-50.0).

[7] Betaxolol: Used in Kim *et al.* (2020) [3] with a dosage of 10mg.

[8] Landiolol: Used in Sasaki *et al.* (2020) [13] with dosages of 1 µg/kg/min and 2 µg/kg/min.

## Dosages and frequencies:

The dosages and frequencies of beta-blocker administration varied across studies:

[1] Metoprolol: Dosages ranged from 5 mg IV to 50 mg orally, with frequencies ranging from twice a day to three times a day.

[2] Carvedilol: Dosage was 12.5mg (3.0-25.0).

[3] Propranolol: Dosage was 20.0mg (20.0-40.0).

[4] Bisoprolol: Dosage was 2.5mg (1.25-5.0).

[5] Nebivolol: Dosage was 2.5mg (1.25-5.0).

[6] Atenolol: Dosage was 50mg (25.0-50.0).

[7] Betaxolol: Dosage was 10mg.

[8] Landiolol: Dosages were 1 µg/kg/min and 2 µg/kg/min.

## Durations of administration:

The durations of beta-blocker administration also varied:

[1] Preoperative administration: Used in studies, including Onk *et al.* (2015) [14] and Puscas *et al.* (2023) [12].

[2] Postoperative administration: Used in studies, including Kim *et al.* (2020) [3] and Skiba *et al.* (2013) [16].

[3] Both pre- and postoperative administration: Used in studies, including Puscas *et al.* (2023) [12] and Halonen *et al.* (2010) [18].

[4] Variable durations: Used in studies, including Cheng *et al.* (2014) [15] and Sasaki *et al.* (2020) [13].

Overall, the drug regimens used in the studies varied significantly, reflecting the lack of standardization in the use of beta-blockers for preventing postoperative atrial fibrillation. Table 3 describes the outcome of studies reporting role of beta-blockers in reducing postoperative atrial fibrillation following cardiac surgery.

## Reduced Incidence of POAF:

[1] Post-operative beta-blocker usage reduced POAF incidence (De Kruijf *et al.* 2024) [11]

[2] Beta-blocker treatment reduced post-operative AF incidence (Puscas *et al.* 2023) [12]

[3] Post-operative BB use reduced POAF occurrence (Kim *et al.* 2020) [3]

[4] Perioperative metoprolol reduced post-operative AF incidence (Skiba *et al.* 2013) [16]

[5] Carvedilol plus NAC decreased POAF incidence (Ozaydin *et al.* 2013) [17]

## No significant difference:

[1] No significant difference in intensive care unit stay, hospital stay, or hospital charges between amiodarone and metoprolol groups (Onk *et al.* 2015) [14].

[2] No significant difference in AF incidence between metoprolol and amiodarone groups (Halonen *et al.* 2010) [18].

## Predictors of POAF:

[1] Age, left ventricular ejection fraction, left atrial diameter, and aortic cross-clamping time predicted post-operative AF (Onk *et al.* 2015) [14].

[2] Age, administration of beta-blocker therapy, and left atrial diameter predicted post-operative AF (Cheng *et al.* 2014) [15]

## Dosage-dependent effects:

[1] Increasing beta-blocker dosage decreased AF incidence (Cheng *et al.* 2014) [15]

[2] Landiolol administration had a trend towards decreased POAF incidence with increasing dose (Sasaki *et al.* 2020) [13]

## Discussion:

The risk factors for post-surgical atrial fibrillation (AF) can be categorized into three main groups: preoperative, intraoperative, and postoperative factors. Preoperative factors include: (a) atrial tissue damage due to age, previous rheumatic fever, elevated left ventricular diastolic pressure, hypertension, and coronary syndromes; [19, 20, 21, 22, 23- 24] (b) heart diseases such as left ventricular hypertrophy, left atrium enlargement, or history of congestive heart failure; [19, 25] and (c) electrolytic imbalance, including hypokalemia, hypomagnesemia, hypothyroidism, and preoperative use of digoxin or milrinone [19, 26]. Additionally, obesity, male gender, chronic obstructive pulmonary disease (COPD), tachycardia, and prolonged P-wave deviation may also predispose to AF [27, 28, 29, 30, 31, 32, 33-34]. Intraoperative risk factors are attributed to increased sympathetic activation due to stimulation of catecholamines, reflex sympathetic activation from volume loss, anemia, pain, adrenergic drug administration, aortic cross-clamping duration, early return of atrial electrical activity after cardioplegia, bicaval venous cannulation, left ventricular venting via pulmonary vein, and extracorporeal circulation [19, 21, 29, 33]. Postoperative AF is correlated with hemodynamic deterioration, including myocardial infarction, heart failure, thromboembolism, and bleeding due to anticoagulation, as well as stroke, hypomagnesemia, prolonged extubation time, increased postoperative P-wave dispersion and exaggerated inflammation reaction [33, 34, 35-36]. Several pharmacological and nonpharmacological strategies have been proposed to prevent atrial fibrillation (AF) after coronary artery bypass grafting (CABG) [37-38]. However, some studies have found that β-blockers may not provide significant benefits in preventing postoperative AF in patients undergoing CABG [37, 38-39]. In contrast, recent evidence suggests that certain medications, including antiplatelet agents, statins, and angiotensin-converting enzyme inhibitors, can reduce the risk of postoperative complications. These medications have been shown to inhibit the inflammatory response triggered by cardiopulmonary bypass (CPB), thereby preventing early arrhythmias, including AF [40, 41-42]. Therefore, effective treatment for the prevention of postoperative AF is crucial. In the present systemic review, studies demonstrated a significant reduction in the incidence of postoperative atrial fibrillation (POAF) with the use of beta-blockers. Specifically, post-operative beta-blocker usage was found to reduce POAF incidence [11]. Similarly, beta-blocker treatment was shown to reduce post-operative AF incidence, [12] and post-operative BB use reduced POAF occurrence [3]. Additionally, perioperative metoprolol was found to reduce post-operative AF incidence 16 and carvedilol plus NAC decreased POAF incidence [17]. Corresponding to present analysis, Norhayati *et al.* [43] conducted a meta-analysis of nine trials involving 1570 patients found that metoprolol significantly reduced the risk of postoperative atrial fibrillation (POAF) compared to placebo, with a relative risk (RR) of 0.46 (95% CI 0.33-0.66) and a risk difference (RD) of -0.19 (95% CI -0.28 to -0.10). However, metoprolol was found to increase the risk of POAF compared to carvedilol, with an RR of 1.59 (95% CI 1.20-2.12) and an RD of 0.13 (95% CI 0.06-0.20). No significant differences were found when metoprolol was compared to sotalol or amiodarone. Additionally, there were no significant differences in cardiovascular conditions or mortality between the groups. Another comparable meta-analysis conducted Zeinah *et al.* [44] of randomized controlled trials (RCTs) provided a clinically useful summary of the results on the prevention of postoperative atrial fibrillation (POAF). The final analysis included 16 trials with 4727 subjects. The network estimates showed that betaxolol, carvedilol, and sotalol were more effective than propranolol, metoprolol, and atenolol in reducing POAF incidence. Notably, carvedilol had the best safety profile, with the lowest risk of patient dropout due to side effects. The key findings indicated that betaxolol (OR 0.36; 95%CI 0.25-0.52), carvedilol (OR 0.36; 95%CI 0.23-0.58), and sotalol (OR 0.38; 95%CI 0.30-0.50) were effective in preventing POAF. In comparison, propranolol (OR 0.51; 95%CI 0.27-0.95), metoprolol (OR 0.72; 95%CI 0.58-0.90), and atenolol (OR 0.81; 95%CI 0.42-1.56) had lower efficacy rates. In conclusion, carvedilol was found to be effective in preventing POAF while maintaining a good safety profile. The overall quality of evidence was moderate to high. The study concludes that metoprolol is effective in preventing POAF compared to placebo, but has a higher risk of POAF compared to carvedilol. Although beta-blockers are widely used to treat cardiovascular diseases, including atrial and ventricular arrhythmias, hypertension, and heart failure, the exact mechanism by which metoprolol reduces the incidence of atrial fibrillation (AF) remains unclear [15]. Research suggests that metoprolol's antiarrhythmic effects may be attributed to its influence on the remodeling of connexin43 (Cx43), a crucial protein for intercellular electrical communication in the heart [45]. Additionally, Dhein *et al.* [46] demonstrated that metoprolol can counteract alterations in Cx43 localization and conduction changes, which may aid in the development of novel antiarrhythmic drugs that modulate gap junction remodeling. The risk of developing postoperative atrial fibrillation (POAF) follows a nonlinear trend, with the highest risk observed on the second postoperative day. POAF risk can be categorized into two distinct phases, each with its own set of risk factors. The initial phase of risk occurs immediately after surgery and decreases sharply within the first 18 hours. Advanced age, longer aortic cross-clamp time and mitral valve surgery are associated with this phase. In contrast, the second phase of risk peaks between 36-48 hours postoperatively and is linked to factors such as advanced age, greater weight, Caucasian race, and mitral valve surgery [47]. Analysing the various studies, it was found that De Kruijf *et al.* (2024) [11] conducted a study involving patients who underwent coronary artery bypass grafting, valve surgery, or a combination of both. The results showed that 135 patients (33%) developed POAF, and pre- and post-operative beta-blockers were administered. Puscas *et al.* (2023) [12] investigated the incidence of POAF in patients undergoing aortic valve replacement, mitral valve replacement, surgical revascularization, or their combination. The study found that 36% of patients experienced postoperative AF, and beta-blockers were used as preoperative, postoperative, or both pre- and postoperative treatment.

Kim *et al.* (2020) [3] conducted a study on patients who underwent aortic valve replacement (AVR) and found that 154 patients (52.0%) developed POAF at a mean of 2.8±1.8 days after surgery. The study examined the usage of beta-blockers, including carvedilol, propranolol, bisoprolol, nebivolol, atenolol and betaxolol, with varying dosages. Sasaki *et al.* (2020) [13] investigated the incidence of POAF in patients undergoing valve surgery, CABG, aorta surgery, or TEVAR. The study found that POAF occurred in 24.4% of patients in the control group, 18.2% in the 1γ group, and 11.1% in the 2γ group. The study used landiolol at different dosages (1 µg/kg/min or 2 µg/kg/min) or no landiolol. Onk *et al.* (2015) [14] conducted a study on patients undergoing coronary artery bypass grafting (CABG) surgery and found that AF developed in 14 patients in the amiodarone group and 16 patients in the metoprolol group 4 weeks after the operation. The study used amiodarone (200 mg/day) and metoprolol (50 mg/day) with varying frequencies and durations. Cheng *et al.* (2014) [15] investigated the incidence of POAF in patients undergoing coronary artery bypass graft (CABG) and/or valve surgery. The study found that the incidence of POAF decreased with increasing dosages of metoprolol, with 33.7% of patients developing POAF without beta-blockers, 26.4% with low-dose beta-blockers (<12.5 mg), 21.5% with medium-dose beta-blockers (12.5-20 mg), and 12% with high-dose beta-blockers (>20 mg). Skiba *et al.* (2013) [16] conducted a study on patients undergoing elective cardiac surgery and used metoprolol (5 mg IV over 5 min at bypass commencement, then 5 mg IV qid for 24 h, then 25-50 mg orally tds until discharge) with or without amiodarone. Halonen *et al.* (2010) [18] investigated the incidence of POAF in patients undergoing first on-pump CABG surgery, aortic valve replacement, or combined CABG and aortic valve surgery. The study found that AF occurred in 23.9% of patients in the metoprolol group and 24.8% of patients in the amiodarone group. The study used metoprolol (1-3 mg/h) for 48 hours and amiodarone (15 mg/kg/day, max 1000 mg/day) starting 15-21 hours after cardiac surgery. Sivapathasundharam *et al.* [48] reported that Type I lip print patterns were predominant among females and Type III among males in an Indian population, suggesting their forensic utility in sex determination. Singh *et al.* [49] demonstrated significant inheritance of lip print patterns, particularly in one quadrant, indicating genetic influence in cheiloscopic features. Kaur *et al.* [50] introduced a scoring system to quantify lip print grooves, confirming the uniqueness of lip prints and their value in individual identification. Rajguru *et al.* [51] found no matching lip print patterns among participants, reinforcing the individuality of lip prints for forensic identification. The studies examined in this review have several limitations. One major limitation is the heterogeneity in study design, patient population, and beta-blocker regimens, which makes it challenging to draw definitive conclusions. Additionally, some studies had small sample sizes, which may limit the generalizability of the findings. Furthermore, the definition and diagnosis of postoperative atrial fibrillation (POAF) varied across studies, which may lead to inconsistencies in reported incidence rates. Moreover, some studies did not control for potential confounding variables, such as underlying cardiac disease or concomitant medications, which may impact the effectiveness of beta-blocker therapy. Finally, the majority of studies focused on short-term outcomes, and the long-term effects of beta-blocker therapy on POAF incidence and clinical outcomes remain unclear.

## Conclusion:

Beta-blockers, particularly when administered postoperatively, significantly reduce the incidence of POAF following cardiac surgery, despite variability in drug regimens. Predictors such as age left atrial diameter and cross-clamp time influence outcomes. Further studies are needed to determine optimal dosing strategies and patient selection.

## Figures and Tables

**Table 1:** Baseline details of studies reporting role of beta-blockers in reducing postoperative atrial fibrillation following cardiac surgery

**Author**	**Year**	**Type of study**	**Country**	**No. of participants in study group**	**Mean age**	**Gender**
De Kruijf N.L *et al.* [11]	2024	Retrospective study	Netherlands	415	-	-
Puscas *et al.* [12]	2023	Cohort study	Europe	472	-	-
Kim *et al.* [3]	2020	Retrospective study	Korea	296 patients	67±12 years.	male:163, Females:133
Sasaki *et al.* [13]	2020	Single-center prospective randomized control study	Japan	150	3 groups: 74.5 ± 3.9, 76.6 ± 3.5, 75.9 ± 5.0	Males:97, Females:53
Onk *et al.* [14]	2015	Prospective randomized study	Turkey	251 patient	2 groups: 56.1 ± 4.1, 59.2 ± 4.2	147 male, 104 female
Cheng *et al.* [15]	2014	Prospective randomized controlled study	China	614	2 groups: 63.11 ± 8.11, 62.93 ± 7.68	342 male, 272 female
Skiba *et al.* [16]	2013	Prospective, randomised, single-blind, controlled pilot study	Australia	215	-	170 male, 45 female)
Ozaydin *et al.* [17]	2013	Prospective randomized study	Turkey	311	-	-
Halonen *et al.*[18]	2010	Randomized, prospective, equivalence, open-label, multicenter study.	Finland	316 consecutive patients who were hemodynamically stable and free of mechanical ventilation and AF within 24 hours after cardiac surgery.	-	-

**Table 2:** Evaluation of studies reporting role of beta-blockers in reducing postoperative atrial fibrillation following cardiac surgery

**Author**	**Operative procedure**	**Postoperative atrial fibrillation (POAF)**	**Drug regimen**
De Kruijf *et al.* (2024)[11]	Coronary artery bypass grafting, valve surgery or a combination of both	135 (33%)	Pre- and post-operative beta-blockers
Puscas *et al.* (2023) [12]	Aortic valve replacement, mitral valve replacement, surgical revascularization, or their combination	36% of patients experienced postoperative AF.	Beta-blockers were used as preoperative (n = 44), postoperative (n = 54) and both pre- and postoperative (n = 175) treatment.
Kim SH *et al.* (2020)[3]	Aortic valve replacement (AVR).	Incidence of POAF: 154 patients (52.0%) developed POAF at a mean of 2.8±1.8 days after surgery.	Beta Blocker (BB) Usage: Total: 296 BB Users: 180 (60.8%) Types and Dosage: Non-selective:- Carvedilol: 54 (30.0%), 12.5mg (3.0-25.0)- Propranolol: 3 (1.7%), 20.0mg (20.0-40.0) β1-selective: - Bisoprolol: 100 (55.6%), 2.5mg (1.25-5.0)- Nebivolol: 17 (9.4%), 2.5mg (1.25-5.0)- Atenolol: 5 (2.8%), 50mg (25.0-50.0)- Betaxolol: 1 (0.6%), 10mg
Sasaki *et al.* (2020) [13]	Valve surgery CABG (Coronary Artery Bypass Grafting) Aorta surgery TEVAR (Thoracic Endovascular Aortic Repair)	POAF occurred in:- 24.4% of patients in the control group.- 18.2% in the 1γ group.- 11.1% in the 2γ group (p = 0.256).	1γ group (landiolol at 1 µg/kg/min), 2γ group (landiolol at 2 µg/kg/min), or control group (no landiolol).
Onk *et al.* (2015) [14]	Coronary artery bypasses grafting (CABG) surgery.	AF developed in 14 patients in the amiodarone group and 16 patients in the metoprolol group 4 weeks after the operation (P = 0.612).	Group I (Amiodarone) Dose: 200 mg/day Frequency: 3 times a day (3 x 1) Duration: 1 week before coronary bypass surgery and during the postoperative period Group II (Metoprolol) Dose: 50 mg/day Frequency: 2 times a day (2 x 1) Duration: 1 week before surgery and during the postoperative period- Patients received amiodarone or metoprolol orally 1 week before surgery and during the postoperative period.
Cheng *et al.* (2014) [15]	Coronary artery bypass graft (CABG) and/or valve surgery	Beta-Blocker (metoprolol) Dose and AF Incidence: No beta-blockers: 175 patients, 59 (33.7%) with AF. Low-dose beta-blockers (<12.5 mg): 208 patients, 55 (26.4%) with AF. Medium-dose beta-blockers (12.5-20 mg): 251 patients, 28 (21.5%) with AF. High-dose beta-blockers (>20 mg): 50 patients, 12 (12%) with AF	12.5-mg metoprolol, 25-mg metoprolol, 50-mg metoprolol
Skiba *et al.* (2013) [16]	Elective cardiac surgery	Not mentioned	Metoprolol: 5 mg IV over 5 min at bypass commencement, then 5 mg IV qid for 24 h, then 25-50 mg orally tds until discharge; Amiodarone + Metoprolol: Amiodarone: 300 mg IV over 1 h after bypass commencement, then 900 mg IV over 24 h, then 400 mg orally tds until discharge; Metoprolol: same as above
Halonen *et al.* (2010) [18]	First on-pump CABG surgery, aortic valve replacement, or combined CABG and aortic valve surgery.	AF occurred in: 38/159 (23.9%) patients in the metoprolol group. 39/157 (24.8%) patients in the amiodarone group (P = 0.85).	Metoprolol (1-3 mg/h) for 48 hours. Amiodarone (15 mg/kg/day, max 1000 mg/day) starting 15-21 hours after cardiac surgery.

**Table 3:** Outcome of studies reporting role of beta-blockers in reducing postoperative atrial fibrillation following cardiac surgery

**Author**	**Outcome**
De Kruijf *et al.* (2024) [11]	"Preoperative beta-blockers' usage was not associated with lower ePoAF prevalence (35% vs 27%). Post-operative beta-blockers' usage was significantly associated with lower ePoAF prevalence (20% vs 61%). De novo ePoAF occurred in 22% of patients using both pre- and post-operative beta-blockers."
	Epicardial mapping data showed that patients with ePoAF had: More low voltage areas and conduction block (CB). More long doubles and fractionated potentials. Less single potentials
	"Post-operative beta-blockers, but not preoperative beta-blockers, are effective in preventing de novo ePoAF. This therapy is only moderately effective in preventing ePoAF. Patients with ePoAF have more extensive arrhythmogenic substrates prior to cardiac surgery."
Puscas *et al.* (2023) [12]	Beta-blocker treatment significantly reduced postoperative AF incidence (p < 0.05). One-month post-surgery, beta-blocker treatment maintained sinus rhythm (p = 0.0001).
	Risk factors for postoperative AF: - Age (p = 0.006)- Left atrium (LA) size (p = 0.006)
	Elevated C-reactive protein (CRP) levels on the fifth postoperative day in patients with AF (p < 0.007). Longer duration of ischemia in patients with AF (p = 0.009). Perioperative beta-blocker treatment is effective in mitigating postoperative AF.
Kim *et al.* (2020) [3]	Postoperative BB use: Significantly reduced the occurrence of POAF (OR, 0.343; 95% CI, 0.163 to 0.724; P=0.005).
	Class of BB and preoperative BB use: No significant association with POAF.
	Other factors associated with POAF: Age, history of stroke, type of aortic prosthesis, chronic kidney disease, and body surface area.
	Multivariable logistic regression model: Postoperative BB use within 24 hours after surgery was a preventive factor of POAF (OR, 0.354; 95% CI, 0.163 to 0.770; P=0.009).
	Age as a risk factor: Significantly associated with the occurrence of POAF (OR, 1.056; 95% CI, 1.032 to 1.081; P<0.001).
Sasaki *et al.* (2020) [13]	Multivariate logistic regression analysis showed a trend towards decreased POAF incidence with increasing landiolol dose (trend-p = 0.120).
	"Subgroup analysis: Significant dose-dependent reduction in POAF among: Female patients (trend-p = 0.02). Patients not using angiotensin II receptor blockers (ARBs) preoperatively (trend-p = 0.03). Patients after valvular surgery (trend-p = 0.004)."
	Prophylactic administration of low-dose landiolol may not be effective for preventing POAF in overall patients after cardiovascular surgery. However, landiolol administration could be beneficial for female patients, patients not using ARBs preoperatively, and those after valvular surgery.
Onk *et al.* (2015) [14]	"Postoperative Outcomes: No significant difference in intensive care unit stays between groups. No significant difference in hospital stays between groups. Hospital charges were similar in both groups (P = 0.741)."
	Predictors of Postoperative AF: Age Left ventricular ejection fraction Left atrial diameter Aortic cross-clamping time Amiodarone and metoprolol have similar effects in preventing AF after cardiac surgery.
Cheng *et al.* (2014) [15]	Factors influencing postoperative AF outcome: Age (OR = 1.04, 95% CI = 1.02-1.07, P = .001) Administration of beta-blocker therapy (OR = 0.57, 95% CI = 0.38-0.84, P = .004)
	Beta-Blocker Dosage and AF Incidence Increase in beta-blocker dosage decreased AF incidence.
	Significant reductions in AF incidence with: 25 mg metoprolol (OR = 0.56, 95% CI = 0.93-0.34, P = .025) 50 mg metoprolol (OR = 0.33, 95% CI = 0.63-0.18, P = .001)
Skiba *et al.* (2013) [16]	No significant difference in AF incidence between groups in intention-to-treat analysis. Per-protocol analysis showed a significant reduction in AF incidence with metoprolol (OR 0.31, p=0.048) but not with amiodarone. High incidence of side effects, especially bradycardia, with perioperative administration of metoprolol and combination of metoprolol with amiodarone. Perioperative metoprolol reduces postoperative AF incidence.
	Combination of metoprolol with amiodarone does not provide additional benefit.
Ozaydin *et al.* (2013) [17]	"POAF incidence was lower in the carvedilol plus NAC group compared to the metoprolol group (P < 0.0001) and the carvedilol group (P = 0.03). Carvedilol group had borderline significance for lower POAF rates compared to the metoprolol group (P = 0.06). Duration of hospitalization was lower in the carvedilol plus NAC group compared to the metoprolol group (P = 0.004)."
	"Multivariate analysis: Independent predictors of POAF included: Left-atrial diameter Hypertension Bypass duration Pre-randomization and pre-operative heart rates, Carvedilol plus NAC group vs. metoprolol group, Carvedilol plus NAC group vs. carvedilol group"
	Carvedilol plus NAC decreased POAF incidence and duration of hospitalization compared to metoprolol. Carvedilol plus NAC decreased POAF incidence compared to carvedilol.
Halonen J *et al.* (2010) [18]	Occurrence of AF similar in metoprolol and amiodarone groups.
